# Case of Foreign Body in the Ear

**Published:** 1873-03-01

**Authors:** D. J. McMillan

**Affiliations:** Biggsville, Ill.


### A CASE OF FOREIGN BODY IN THE EAR.

BY D. J. McMILLAN, M.D., BIGGSVILLE, ILL.

The Rev. Crawford, who was visiting at my house, with family, requested me to examine his little girl's ear for some foreign substance, which was seen, and supposed to be a grain of pop-corn. The history given was this: The child (four years old last October) while playing with some pop-corn, three years and four months ago, put several kernels in the left ear. Several unpopped grains were removed at the time, and, it was supposed, all of them.

Nothing more was thought of the matter until about a year after. At this time the child had the whooping-cough; and after a coughing spell, on looking into the ear, something resembling a grain of pop-corn was seen.

Drs. Heller and Reese, of Abingdon, were sent for. Before they came, however, the foreign substance had disappeared, and the doctors, not being able to discover anything, came to the conclusion that if anything had been there, it was thrown out in a fit of coughing.

Time passed on until about six weeks ago. Some shining substance again made its appearance in the external meatus. A strong suspicion was again aroused that this was the veritable grain of corn supposed to have been left in the ear over three years previous. This suspicion was the more plausible from the fact that, since the accident first occurred, the child had been greatly afflicted with the earache; and of late marked deafness had supervened, although no discharge had occurred, and the deafness and earache had seemingly been the same in both ears.

Dr. McDill, of this place, was called in. With the aid of a speculum he discovered a hard substance, deep in the cavity of the ear; but owing to the nervous irritability of the little patient, failed in extracting whatever it was. He ordered the ear to be syringed, hoping by this means to dislodge it. The syringing was followed as directed; and frequently the shining substance could be seen, but not expelled. It was at this stage of the case that I was requested to examine the ear. On examination, found a hard, shining body midway between the tympanum and the opening of the external meatus; and finding it impossible to get the child still enough to extract with instruments, concluded to try the plan of Dr. Gowenberg, of Paris, as detailed in the September number of The Examiner. A small brush was made by rolling two layers of thin, old muslin on a match, letting the muslin project the eighth of an inch over the end, and raveling the free edge out up to the match, after it was fastened by a thread. The patient was then got ready by placing in a cradle, with the affected ear up, and in such an inclination as to let the light fall on the object. The glue being heated to liquid consistency, the point of the brush was dipped so as to retain but a small amount of the glue, and then gently passed down the cavity of the ear until the object was felt, and left in that position for three-quarters of an hour. At the end of that time the brush was gently pulled in the axis of the external opening of the ear until withdrawn. A hard, rounded substance remained firmly attached to the glue on the end of the brush, which, after due inspection, proved to be a discolored grain of pop-corn, which the parents firmly believe had been in the ear three years and four months. They (the parents) are confident the child has not had any chance to get the corn in the ear since the time spoken of, over three years previous. What is singular, is the corn remaining such a length of time without generating, or decaying in some way, or producing inflammation and suppuration, and thus being expelled by nature's process.

